# Supplementary material for: Attaining the Promise of Geminivirus-Based Vectors in Plant Genome Editing
Source: Viruses. 2025 Apr 27;17(5):631. doi: 10.3390/v17050631 (PMC12116149; doi:10.3390/v17050631)
Supplement: Supplementary file 1 [file viruses-17-00631-s001.zip › viruses-3524076-supplementary.pdf]

Table S1. List of geminiviruses, their genomic composition and viral vector approach deployed in genome editing.

Virus	Genus	Mono- or bipartite	Genome size	Genes on complementary-sense strand	Functions of C-sense strand proteins	Genes on virion-sense strand	Functions of V-sense strand proteins	Viral vector approach	Reference
BeYDV	*Mastrevirus*	monopartite	2.6-2.8 kb	C1 and C2	Viral DNA replication; replication initiator protein	V1 and V2	Coat protein and movement protein. These proteins are also important for the spread of virus and symptoms development.	**1.** Replaced coat and movement proteins with donor DNA and nucleases.	[1,2]
WDV								**1.** Replaced V1 and V2 with a reporter gene	
MSV								**1.** Replaced V1 and V2 with *CAT* or the *Hph* genes in maize plants.<br>**2.** *Ds1* (405 bp long transposable element) is inserted into SIR.<br>**3.** *GUS* gene is inserted into SIR – virus still able to replicate but unable to induce viral symptoms. Whereas replacing V1 and V2 with GUS was more robust.	[3–5]
CaLCuV	*Begomovirus*	bipartite (DNA-A and DNA-B)	each genome is 2.5 - 2.6 kb long	**DNA-A:** C-sense strand encodes AC1, AC2, AC3 and AC4<br><br>**DNA-B:** BC1 - movement protein	Viral DNA replication; TrAP transactivates expression of V-sense gene expression from DNA-A and DNA-B; involved in the	**DNA-A:** virion-sense strand encodes AV1 and AV2	V1: encapsidates the virion-sense ssDNA, involved in virus movement	**1.** Replaced coat protein with U6 promoter and gRNA.<br>**2.** CaLCuV was used as a full virus vector to insert 18 bp sequence within the *ADH1* in *A. thaliana*.	[1,6]

					suppression of transcriptional and post-transcriptional gene silencing; symptoms determinant implicated in cell-cycle control	**DNA-B:** BV1-encodes a nuclear shuttle protein			
SPLCV		monopartite	2.6-2.8 kb	C1, C2, C3 and C4	Required for initiation of viral DNA replication, C2 acts as a pathogenicity factor in some hosts, C3 and C4 – important for system determinant implicated in cell-cycle control.	AV1 and AV2	Coat protein and movement protein	Replaced AV1 and AV2 genes with GFP expression cassette.	[7]
BCTV	*Curtovirus*	monopartite	2.9-3.0 kb	C1, C2, C3 and C4		V1, V2 and V3	V1: encapsidates the virion-sense ssDNA, involved in virus movement, insect transmission,<br>V2 acts as a suppressor of host silencing and is essential for systemic infection and, V3 is involved in the regulation of the relative levels of ssDNA and dsDNA.	**1.**Virion-sense genes have been replaced with heterologous promoters. E.g., 35S promoter upstream of the Cas12a and other cargoes.<br>**2.** utilized to induce gene silencing and for GOI insertion in the antisense orientation downstream of a truncated V-sense genes.<br>**3.** removing of V-sense genes including V2 and V3 enhanced cargo expression while for GT, ssDNA replicon containing V-sense genes performed better.	[8]
